# The specific and combined role of domestic violence and mental health disorders during pregnancy on new-born health

**DOI:** 10.1186/s12884-017-1438-x

**Published:** 2017-08-01

**Authors:** Alexandre Archanjo Ferraro, Luis Augusto Rohde, Guilherme Vanoni Polanczyk, Adriana Argeu, Euripides Constantino Miguel, Sandra Josefina Ferraz Ellero Grisi, Bacy Fleitlich-Bilyk

**Affiliations:** 1 Department of Pediatrics, School of Medicine, Universidade de Sao Paulo, Av Dr Eneas Carvalho Aguiar, 647, Sao Paulo, 05403-900 Brazil; 2 Department of Psychiatry, School of Medicine, Universidade de Sao Paulo, Sao Paulo, Brazil; 3 Department of Psychiatry and Legal Medicine, School of Medicine, Universidade Federal do Rio Grande do Sul, Porto Alegre, Brazil

**Keywords:** Domestic violence, Mental health disorders, Birth weight, Birth length

## Abstract

**Background:**

Addressing impaired foetal growth is recognized as a public health priority. Certain risk factors for this condition, such as poor nutritional status at birth, have been found to be highly correlated with poverty. However, the role of psychosocial factors, specifically the mother’s mental health and exposure to violence during pregnancy, have yet to be further explored. Our objective was to determine if there is a measurable association between combined psychosocial factors, specifically domestic violence and mental disorders, and birth outcomes, specifically birth nutritional status and preterm delivery.

**Methods:**

We followed 775 women from an underserved, urban area, beginning their 28th week of gestation. Diagnostic interviews were performed to determine if any of the mothers had any of the following disorders: mood disorder, anxiety, obsessive–compulsive disorder (OCD), substance dependence, psychotic disorder, or anti-social personality disorder. Physical, psychological, and sexual domestic violence were also assessed.

**Results:**

Domestic violence and mental disorders were highly correlated in our sample. About 27.15% of the women in our study experienced domestic violence, and about 38.24% of them were diagnosed with mental disorders. The main association we found between combined psychosocial factors and neonate outcomes was between anxiety (IRR = 1.83; 95%CI = 1.06–3.17)/physical violence (IRR = 1.95; 95%CI = 1.11–3.42) and the rate of small-for-gestational age (SGA) in new-borns. More specifically, the combination of anxiety (beta = −0.48; 95%CI = −0.85/−0.10) and sexual violence (beta = −1.58; 95%CI = −2.61/−0.54) was also associated with birth length. Maternal risk behaviours such as smoking, drinking, inadequate prenatal care, and inadequate weight gain could not sufficiently explain these associations, suggesting that these psychosocial factors may be influencing underlying biological mechanisms.

**Conclusion:**

Domestic violence against women and mental disorders amongst pregnant women are extremely prevalent in under-resourced, urban areas and ultimately, have detrimental effects on birth outcomes. It is imperative that actions be taken to prevent violence and improve mental health during pregnancy.

**Electronic supplementary material:**

The online version of this article (doi:10.1186/s12884-017-1438-x) contains supplementary material, which is available to authorized users.

## Background

Around eighteen million babies worldwide are born with low birth weight (LBW) every year – constituting 14% of all yearly births. Addressing impaired foetal growth and development, such as LBW, is recognized as a key public health priority because there are clear links between LBW and adverse outcomes later in life. For example, children born with LBW have higher chances of developing diabetes, hypertension, stroke, obesity, and mental health disorders as adults [1, 2]. Birth size largely reflects the quality of the intrauterine environment, which in turn, reflects the mother’s environment during the child’s early development [3]. For example, birth weight is highly influenced by the mother’s nutritional status, which in turn, is highly influenced by family income, maternal education, health-seeking attitudes of the family, feeding practices, and ‘risky’ environments [4]. Although a strong consensus exists in support of the relationship between nutrition and birth outcome, the role of psychosocial factors has yet to be further explored. In the last decade, both maternal experience of violence and mental health, mainly depression, have been linked with threats to the health of a child [5–8] and further research should be conducted to deepen our understanding of their underlying mechanisms.

A meta-analysis of 14 published studies from developed countries found a small but significant effect of violence during pregnancy on low birth weight (OR = 1.4; 95% CI 1.1–1.8) [9]. However, few studies of this sort have been carried out in developing countries. In a review of all research concerning the relationship between psychosocial factors and pregnancy outcomes, Paarlberg et al. [10] concluded that “studies on the association between maternal stressor exposure and birth weight have yielded mixed results” and thus, a firm conclusion could not be drawn. However, these psychosocial factors have generally been studied separately, and have remained limited in the number of mental disorders covered. This is an important research question because, if a connection between maternal psychological stress/mental disorders and specific developmental neonate outcomes is established, then preventative actions in clinical practice and public health efforts can be taken to ameliorate their effects.

The aim of this work is to measure the association between domestic violence and different mental disorders during pregnancy and neonate outcomes in a middle-income country. Our hypothesis is that these psychosocial stressors are linked to negative birth outcomes; that their combined presence will result in increased negative birth outcomes; and that the pathway in which they act is by influencing maternal risky behaviours during pregnancy.

## Methods

### Study design

The Butantan birth cohort is a population-based birth cohort following mothers from their 28th week of gestation to the present. The present analysis used data from the T0 (28th gestational week) and T1 (2nd month after birth) phases.

### Population and sample

All pregnant women who were attending pre-natal care in 5 primary care facilities in the region of Butantan – a health district in the western region of the city of Sao Paulo, Brazil – from July 2010 to December 2012, were eligible to enrol in the study. Although this area receives good pre-natal care coverage in the form of monthly home visits through the Family Health Strategy [11], the region is considered an area of great social vulnerability. The region is far from the city centre, is densely populated, and consists of mostly poor families along with a few, newly formed lower-middle income class families. Violence, such as constant encounters between drug dealers and police, co-exists with nurseries, schools, and churches.

Although theoretically, all eligible mothers could have participated in our study, we only included the first 5 eligible subjects assessed per week from each primary health facility. The criterion for eligibility included residence in the area described. Twins and children who were born with any disease associated with impaired development were excluded from analysis.

The planned sample size of the study was 900 women. This size would produce a statistical power of 90% in identifying an association between depression and LBW – assuming a 20% frequency of depression, a 9% incidence of low birth weight, and a relative risk of 1.80.

### Variables

The outcome variables included birth weight (BW), birth length (BL); being born small for gestational age (SGA); and preterm birth (PTB). BW (g) and BL (cm) were outcomes treated as continuous variables. This information was measured according to routine hospital protocol and collected from the clinical reports taken on the birth. SGA was determined according to the Williams reference curve [12]. Gestational age was evaluated using ultra sound: 51.79% of the sample had the ultra sound performed by their 12th week of gestation, while the rest of the mothers did not have an ultra sound until after this period. In this later case, gestational age was based on the mother’s reported last menstrual period or through the Capurro Somatic Method [13], which was assessed by a paediatrician at birth. The Capurro Somatic Method was used in cases where the gestational age determined by the ultrasound differed by more than 2 weeks from the gestational age determined by the last reported menstrual period. All women delivered in maternity hospitals.

Trained psychologists began data collection in the beginning of the third trimester – around the 28th week – during routine pre-natal care visits. Domestic violence against women was measured according to the WHO Domestic Violence Questionnaire [14]. Questions 704 to 706 in the Questionnaire assess for 7 types of physical abuse, 4 types of psychological abuse, and 3 types of sexual abuse perpetrated by the husband/partner in the last 12 months. Given that the mothers were in their 6th month of gestation when assessed, violence may have occurred immediately before or during gestation (or, in some cases, during both these periods). Mental health disorders were measured according to the Mini International Neuropsychiatric Interview (MINI) – a short and structured diagnostic interview used to reveal current disorders [15]. Both instruments had been previously adapted to the Brazilian population before being used in this study [16, 17]. Physical, psychological, and sexual violence were analyzed separately and then pooled in one variable named “violence”. Recurrent episodes of domestic violence reported within the last 12 months were considered positive. Mental disorders were pooled in the following groups: “*mood disorder*” – current hypomanic episode (ICD-10 F31.8), current manic episode (ICD-10 F30.x), current dysthymic disorder (ICD-10 F34.1), current major depression (ICD-10 F32.x); “*anxiety*” – current panic disorder without agoraphobia (ICD-10 F40.01), current panic disorder with agoraphobia (ICD-10 F40.0), social phobia (ICD-10 F40.1), post-traumatic stress disorder (ICD-10 F43.1), general anxiety disorder (ICD-10 F41.1); “*OCD-obsessive compulsive disorder*” – (ICD-10 F42.8); “*substance dependence*” – alcohol and illicit drugs dependence (ICD-10 F10.2× and F11.0 - F19.1), “*anti social personality disorder*” – (ICD-10 F60.2), and “*psychotic disorder*” – (ICD-10 F32.3 and F33.3).

Professionals were trained according to the WHO guidelines presented in “Putting Women First: ethical and safety recommendations for research on domestic violence against women” [18]. The training also focused on how to cope with the challenges of such field-work. Women who were diagnosed with a mental disorder were advised to refer to their general practitioners. Those who were identified as victims of domestic violence were advised to look for the closest Center for Victims of Domestic Violence. Field researchers only directly referred cases to local health facilities (doctor, nurse or social worker) in high risk cases, in which it had been concluded that the woman was unable to follow the previous advice.

Other independent variables were family socioeconomic status (according to the Brazilian Association of Population Studies – ABEP [19], where “A” is the wealthiest and “E” the poorest), years of maternal schooling (stratified into three categories: 0–7 years, 8–10 years, and 11 or more years of completed education – where federal mandatory schooling is 8 years), occupation of the father of the offspring (based on the International Classification of Occupation [20] and classified as “*non-manual*” or “*qualified, semi-qualified*” and “*unskilled manual*”), maternal age (adolescent or non-adolescent mother), maternal migration (being born in Sao Paulo or not), birth order (categorized as primipara or not), reported drinking and smoking habits during gestation (yes or no), unwanted pregnancy (negative feelings towards pregnancy, having considered an abortion – yes or no), sex of the offspring (male or female), number of prenatal care visits (classified as adequate or inadequate for the gestational age), and gestational weight gain - a proxy of maternal nutritional habits (classified as adequate or inadequate for the gestational age, taking into account the pre-gestational body mass index - BMI). These last two variables were classified according to the standards provided by the Brazilian Ministry of Health.

### Data bases and analysis

Data was collected on paper and later transferred to an Excel database (version 6.01). Double entry and verification of data were incorporated in this process to minimize data input errors. Statistical analysis was done using STATA software (version 10.0). Dichotomous variables had their proportion calculated in %, with a respective 95% confidence interval. Continuous variables had their mean and standard deviation calculated and were checked for outliers (defined as below “1st quartile-1.5 × interquartile interval” and above “3rd quartile + 1.5 × interquartile interval”).

We began our analysis by calculating the loss-to-follow-up rate using the chi-square method to test for any selection bias (Additional file 1: Table S1). Then, the description of the studied sample was determined according to exposure of the psychosocial variables: violence or mental disorders. Next, we tested for associations between each exposure (not specified in their categories, but as pooled information) and the outcomes through bivariate Poisson regression (for binary outcome variables, which produced incidence rate ratios - IRR) and linear regression (for continuous outcome variables, which produced beta scores). Violence and mental disorders were analysed both separately and together, and finally presented in graphs. Models using non-pooled violence and mental disorders were then built. Confounders for which models were adjusted were chosen based on the assumption that they must be antecedent of exposure *and* outcome [21]. Since the violence measurement only encompassed the last 12 months, the independent variables that fulfilled this assumption were maternal schooling, maternal migration, family socioeconomic status, and being an adolescent mother. Finally, we tested whether the associations could be explained by the presence of maternal risk behaviours, namely smoking, drinking, inadequate prenatal care, and inadequate weight gain. Stressful events could increase the likelihood of these behaviours, which in turn could affect foetal development. If the association disappears when these variables are included in the models it means that these variables completely explain the link between mental disorders/domestic violence and neonate outcomes, since they would be in the pathway between exposure and outcome.

Ultimately, the null hypothesis was rejected when the probability of occurring type I error was smaller than 5%.

### Ethics

All mothers who were invited to take part in the study were presented with a term of agreement by a professional trained in clarifying the terms in cases where the mother exhibited difficulty in reading or understanding. Mothers read and signed an informed consent after it was determined that they understood the scope of the project, and data from hospital charts were used only after the mothers’ consent. The local ethics in research committee (CAPPesq) approved this research protocol (research protocol number 0054/09).

## Results

Nine hundred women were assessed during pregnancy and 775 of them were followed up to the T1 phase (puerperium). Seven mothers had twins and one mother had a child with Down syndrome, none of which were included in the present analysis. The other mothers lost in the study (*n* = 117, or 13.1%) resulted from their migration out of the sample neighbourhood and loss of contact after repeated telephone calls and home visits. The subjects that were followed-up did not differ from the original enrolled sample in regards to the occupation of the father of the child, family socioeconomic status, maternal schooling, having an unwanted pregnancy, reported smoking and drinking during gestation (Additional file 1: Table 1), and being an adolescent, migrant, or primipara mother. Six children also died between the 28th week of gestation and the first week of life.

**Table 1:** Description of the sample of pregnant women from the Butantan birth cohort according to their exposure to mental disorder or to domestic violence in the last 12 months

		w/ Mental Disorder (*n* = 296)	no Mental Disorder (*n* = 478)	w/ Abuse (*n* = 205)	no Abuse (*n* = 550)
Variable	Category	% (95%CI)	% (95%CI)	% (95%CI)	% (95%CI)
Paternal occupation	Non-manual	11.90 (0.08–0.16)	20.92 (17.18–24.65)**	12.11 (7.42–16.79)	19.77 (16.34–23.20)*
	Qualified manual	18.22 (13.57–22.86)	18.30 (14.75–21.85)	17.37 (11.93–22.80)	18.62 (15.26–21.97)
	Nonqualified manual	69.89 (64.37–75.41)	60.78 (56.30–65.27)	70.53 (63.98–77.07)	61.61 (57.42–65.80)
Family Economic Class	A + B	13.85 (9.89–17.81)	20.29 (16.67–23.91)**	18.54 (13.17–23.90)	17.82 (14.61–21.03)*
	C	63.85 (58.35–69.35)	66.32 (62.07–70.57)	58.54 (51.74–65.34)	67.45 (63.53–71.38)
	D + E	22.30 (17.53–27.07)	13.39 (10.33–16.45)	22.93 (17.12–28.73)	14.73 (11.76–17.70)
Maternal Schooling	< 8 year	21.62 (16.90–26.34)	17.19 (13.79–20.59)	17.56 (12.31–22.81)	19.31 (16.00–22.62)
	8–10 years	40.20 (34.58–45.82)	37.53 (33.17–41.89)	43.42 (36.57–50.26)	36.25 (32.21–40.28)
	11 or + years	38.18 (32.61–43.74)	45.28 (40.80–49.77)	39.02 (32.29–45.76)	44.44 (40.27–48.61)
Adolescent Childbearing	−	21.28 (16.59–25.97)	21.97 (18.24–25.69)	25.85 (19.81–31.90)	20.90 (17.50–24.32)
Maternal Migration	−	40.13 (34.50–45.77)	46.53 (42.02–51.03)	34.80 (28.21–41.40)	47.17 (42.97–51.36)**
Primipara	−	40.20 (34.58–45.82)	51.15 (46.65–55.66)**	40.00 (33.24–46.76)	50.46 (46.26–54.65)*
Unwanted pregnancy	−	29.15 (23.94–34.37)	13.24 (10.18–16.29)**	29.76 (23.44–36.07)	15.15 (12.14–18.16)**
Smoking in pregnancy	−	23.31 (18.47–28.16)	11.55 (8.67–14.44)**	24.39 (18.46–30.32)	12.96 (10.14–15.78)**
Drinking in pregnancy		10.47 (6.96–13.98)	8.60 (6.07–11.12)	15.12 (10.18–20.07)	7.10 (4.95–9.26)*
Prenatal care	Inadequate n. of visits	36.82 (31.30–42.35)	30.54 (26.40–34.69)	37.07 (30.41–43.74)	31.09 (27.21–34.97)
Gestational weight gain	Inadequate for pre gest BMI	40.50 (33.64–47.36)	45.68 (40.50–50.86)	39.44 (31.30–47.57)	45.05 (40.18–49.92)

One missing data for mental disorder and 20 missing data for domestic violence; inadequate number of visits took into account the gestational age; inadequate gestational weight gain took into account the pre-gestational body mass index (IMC); * *p* < 0.05; ** *p* < 0.01

Most families in our sample belonged to the socioeconomic class C (low middle-income class), and were headed by “manual, non-skilled professionals.” Table 1 shows that pregnant women with mental disorders and victims of domestic violence were more likely to have a partner with a less qualified job, were more likely to come from a lower income class, and were more likely to not be primipara. They also had higher incidences of unwanted pregnancy, and they reported smoking and drinking more than the national average. Furthermore, women who suffered abuse in the study were more likely to have been born in the sample area than they were to have been a migrant to the community.

Violence, in one form or another, was reportedly experienced by 27.15% of the women, while 38.24% exhibited some form of mental disorder. More specifically, psychological, physical, and sexual violence were reported by 24.77%, 13.46% and 2.23% of the women, respectively. Mental disorders were prevalent in the mothers as follow: 29.97% for mood disorders; 16.26% for anxiety disorder; 4.52% for Obsessive Compulsive Disorder (OCD); 4.13% for substance dependence; 3.61% for psychotic disorder and 2.19% for anti-social personality disorder.

Among the 775 neonates, 47.95% were born male, 9.29% were born small for gestational age (SGA), 5.81% were born low birth weight (LBW) and 7.11% were born premature. Hypoxia was present in 7.47% of the new-borns. Averages for weight and length (sd) were 3221 (492) and 48.49 (2.04), respectively.

**Fig. 1:**
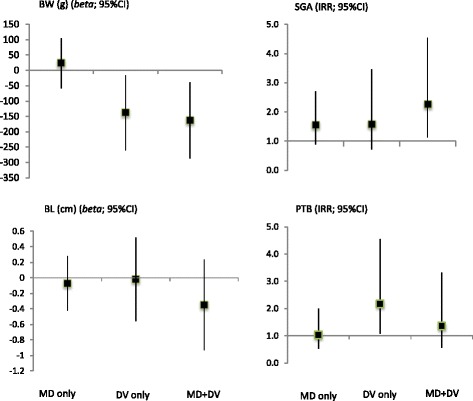
Effect Size of the Association of Pooled Mental Disorders and Pooled Violence with Birth Outcomes

Domestic violence against women was highly linked to mental disorders during gestation. Among women who reported having experienced violence in the last 12 months, 62.9% (*p* < 0.001) them were diagnosed with a mental disorder, while only 28.7% of women who did not report experiencing violence were diagnosed. Figure 1 shows the unadjusted effect size of the association of pooled mental disorders and pooled violence with birth outcomes. Birth weight was statistically associated with domestic violence (DV) (*beta =* −138.08; 95% CI -260.45 / -15.71) and presented a stronger association with mental disorders (MD) plus DV (*beta* = −163.01; 95% CI -287.51 / -38.51). Birth length had no association. Being exposed to both MD and DV increased the likelihood of being SGA (IRR = 2.25; 95% CI 1.12–4.54). Moreover, DV alone increased the risk of preterm birth (2.17; 95% CI 1.04–4.54).

Tables 2 and 3 show the unadjusted and adjusted analysis of the association between all types of violence and mental disorders on birth outcomes. SGA was associated with physical violence, anxiety, and OCD – these first two factors are shown in the unadjusted and adjusted analysis, while the latter is shown only in the unadjusted analysis. Anti-social personality disorder doubled the risk for preterm birth. This finding takes into account that there were only 4 women that presented these exposures and outcomes together, and therefore, serves as an estimate with large confidence intervals. Birth weight was associated with physical and sexual violence in both unadjusted and adjusted analyses, and anxiety disorder presented a tendency towards significance (*p* = 0.067 in the adjusted model). Finally, length was associated with sexual violence and anxiety disorder in both the unadjusted and adjusted analyses.

**Table 2:** Bi and multivariate regression analysis between current mental disorders in pregnancy and birth outcomes in the Butantan birth cohort

Birth Outcome	Unadjusted Models	p	Adjusted Models^a^	p	p*
SGA	IRR (95%CI)		IRR (95%CI)		
Mood dis	1.52 (0.95–2.45)	0.084	1.40 (0.85–2.29)	0.182	
Anxiety dis	1.72 (1.01–2.93)	0.047	1.83 (1.06–3.17)	0.030	0.023
OCD	2.28 (1.04–4.97)	0.039	2.00 (0.90–4.43)	0.087	
Substance dep	1.37 (0.50–3.74)	0.545	1.23 (0.44–3.40)	0.691	
Psychotic dis	0.38 (0.05–2.70)	0.331	0.36 (0.05–2.61)	0.311	
AS Personality dis	1.94 (0.61–6.16)	0.262	1.65 (0.51–5.38)	0.407	
PTB					
Mood dis	0.96 (0.53–1.72)	0.881	1.06 (0.58–1.92)	0.854	
Anxiety dis	0.76 (0.35–1.69)	0.506	0.81 (0.36–1.82)	0.616	
OCD	0.85 (0.21–3.48)	0.818	0.92 (0.22–3.82)	0.914	
Substance dep	0.88 (0.21–3.59)	0.853	0.93 (0.23–3.84)	0.922	
Psychotic dis	0.49 (0.07–3.57)	0.484	0.55 (0.08–4.03)	0.557	
AS Personality dis	3.50 (1.26–9.68)	0.016	4.62 (1.60–13.35)	0.005	0.003
Weight (g)	beta (95%CI)		beta (95%CI)		
Mood dis	−28.97 (−105.73/47.80)	0.459	−20.46 (−98.38/57.47)	0.606	
Anxiety dis	−80.29 (−175.53/14.95)	0.098	−89.89 (−186.01/6.24)	0.067	
OCD	32.15 (−137.36/201.66)	0.710	51.70 (−118.13/221.52)	0.550	
Substance dep	−82.97 (−259.81/93.87)	0.357	−66.77 (−243.26/109.73)	0.458	
Psychotic dis	92.32 (−96.21/280.85)	0.337	98.73 (−90.60/288.07)	0.306	
AS Personality dis	−184.90 (−424.87/55.08)	0.131	−163.68 (−405.18/77.81)	0.184	
Length (cm)					
Mood dis	−0.04 (−0.34/0.26)	0.795	−0.03 (−0.34/0.28)	0.851	
Anxiety dis	−0.41 (−0.78/−0.04)	0.030	−0.48 (−0.85/−0.10)	0.013	0.017
OCD	−0.20 (−0.89/0.49)	0.566	−0.21 (−0.91/0.48)	0.549	
Substance dep	−0.34 (−1.04/0.36)	0.341	−0.32 (−1.02/0.386)	0.379	
Psychotic dis	0.26 (−0.47/0.99)	0.479	0.24 (−0.49/0.97)	0.518	
AS Personality dis	−0.66 (−1.57/0.24)	0.150	−0.70 (−1.61/0.21)	0.132	

*IRR* incidence rate ratio from Poisson regression analysis; “beta” from linear regression analysis, *95% CI* (95% confidence interval), *SGA* small for gestational age, *PTB* preterm birth, *dis* disorder, *dep* dependence, *OCD* obsessive compulsive disorder, *AS* anti-social

*Same adjustments plus for maternal risk behaviours: smoking during gestation, drinking during gestation, inadequate number of prenatal care visits for the gestational age and inadequate weight gain for the gestational age considering the pre-gestational BMI

^a^Adjusted for socio-demographic variables: maternal schooling, economic class, adolescent childbearing, maternal migration

**Table 3:** Bi and multivariate regression analysis between different types of domestic violence during the last year and birth outcomes in the Butantan birth cohort

Birth Outcome	Unadjusted Models	p	Adjusted Models^a^	p	p*
SGA	IRR (95%CI)		IRR (95%CI)		
Physical violence	2.06 (1.19–3.56)	0.009	1.95 (1.11–3.42)	0.021	0.168
Psychological violence	1.03 (0.60–1.76)	0.910	0.94 (0.54–1.62)	0.816	
Sexual violence	1.96 (0.62–6.25)	0.252	2.13 (0.66–6.82)	0.205	
PTB					
Physical violence	1.27 (0.62–2.60)	0.512	1.30 (0.63–2.70)	0.478	
Psychological violence	1.51 (0.86–2.66)	0.153	1.51 (0.85–2.67)	0.159	
Sexual violence	1.65 (0.40–6.79)	0.485	1.94 (0.47–8.02)	0.362	
Weight (g)	beta (95%CI)		beta (95%CI)		
Physical violence	−166.61 (−269.74/−63.49)	0.002	−148.52 (−252.95/−44.09)	0.005	0.016
Psychological violence	−72.00 (−153.55/9.55)	0.083	−62.25 (−144.05/19.55)	0.136	
Sexual violence	−232.26 (−471.79/7.27)	0.057	−244.78 (−483.72–5.84)	0.045	0.082
Length (cm)					
Physical violence	−0.40 (−.82/0.02)	0.061	−0.34 (−0.76/0.09)	0.122	
Psychological violence	−0.10 (−0.43/0.23)	0.540	−0.06 (−0.39/0.27)	0.715	
Sexual violence	1.53 (−2.56/−0.49)	0.004	−1.58 (−2.61/−0.54)	0.003	0.008

*IRR* incidence rate ratio from Poisson regression analysis; “beta” from linear regression analysis, *95% CI* (95% confidence interval), *SGA* small for gestational age, *PTB* preterm birth, *dis* disorder, *dep* dependence, *OCD* obsessive compulsive disorder, *AS* anti-social

*Same adjustments plus for maternal risk behaviours: smoking during gestation, drinking during gestation, inadequate number of prenatal care visits for the gestational age and inadequate weight gain for the gestational age considering the pre-gestational BMI

^a^Adjusted for socio-demographic variables: maternal schooling, economic class, adolescent childbearing, maternal migration

These findings did not change considerably when maternal risk behaviours were included in the models (Tables 2 and 3). This suggests that the pathway, which explains the association, does not pass exclusively through these behaviours. Ultimately, we could not confirm the hypothesis that the pathway through which mental disorders and violence have an effect on the offspring is by influencing maternal risk behaviours during pregnancy.

## Discussion

Violence is a major public health issue in developing countries, where 90% of these events occur [22]. In Latin America, this has been found to be primarily an urban phenomenon, with the highest concentrations of violence occurring in the peripheries of major cities [23, 24]. The global estimate for repeated intimate partner violence among ever-partnered women is 30.0% (95% CI 27.8–32.2) [25]. A recent meta-analysis found that violence towards women during pregnancy remains at an average of 14.4% (IQR 13.2–25.7) [26]. In developed countries, physical violence against the female partner has been estimated to occur in up to 20% of pregnancies [27]. Meanwhile, in Latin America, a Mexican [28] study reported rates of 33.5%, while a Brazilian study reported a rate of 33.8% [29]. Moreover, maternal mental disorders are also highly prevalent. A systematic review [30] of studies of depression during pregnancy, which included 21 studies (only one from a developing country) reported a prevalence of 7.4%, 12.8% and 12% for the first, second, and third trimesters, respectively. Heron et al. [31] revealed anxiety disorder in 7.3% of mothers in a British population during their third trimester. However, limited data exists from developing countries [32, 33].

This study adds to the literature on violence and mental health during pregnancy by providing a follow-up research study in an underserved, urban population in a middle-income country – a context still vastly underrepresented in studies – and by assessing a larger range of mental health problems than has been done in the past. Detailed assessments measuring psychological stress and evaluating mental disorders were performed using diagnostic interviews and screening scales; high incidences of domestic violence and mental disorders were recorded.

This relation between violence and poor mental health is not new. In a meta-analysis, Golding [34] found that the weighted Odds Ratios of the association between different mental disorders and violence varied from 3.5 to 5.6; the most common disorders among those who suffered violence were depression and post-traumatic stress disorders. A cross-sectional study in Brazil [35] found that adolescents who were victims of violence during pregnancy were 4.3 times more likely to also suffer from common mental disorders (95% CI 1.7–10.9). More recently, the WHO reported a pooled OR = 1.97 (95% CI 1.56–2.48) in a review of 6 studies about the association between depression and violence [25]. Recent trends in urbanization and westernization have been thought to contribute to the rise in violence and mental health problems, as a result of changes in family structure, deterioration of traditional social networks, new environments challenging traditional values and beliefs, and other emerging categories of vulnerability [36]. Moreover, adverse life events, such as violence, are known risk factors for poor mental health [37], thus supporting our finding that the magnitude of negative effects would be even higher when both factors were present.

Although the association we found between violence and mental disorders and neonate outcomes had been confirmed in some parts of the literature, many studies yielded mixed results. However, there is evidence that suggests that this association may only be apparent in communities of lower socioeconomic status. Anderson et al. [6] did not find an association when conducting a study among Swedish women, and neither did Chung et al. [7] among women in Hong Kong. Hoffman et al. [38], on the other hand, found a positive association, but only among women from an under-resourced African-American community in the United States. Rahman et al. [8] also found an independent association between maternal antenatal depression and low birth weight among 632 mothers in rural Pakistan, and Patel & Prince [39] found it in a study of 270 pregnant women in Goa. In Nicaragua [40], Mexico [28], China [41], India [42] and a previous study in Brazil [43] reported negative outcomes as well.

Nonetheless, our findings suggest that a positive association persists even after controlling for socio-economic and demographic confounders (socioeconomic status, maternal schooling, maternal migration and being an adolescent mother). We found that in this under-resourced, urban area, domestic violence and mental health in pregnant women are highly prevalent and intimately correlated. From this study, we can extrapolate that violence (physical and sexual) and anxiety disorder have a negative effect on birth variables, i.e., birth weight, birth length, and the likelihood of being a SGA new-born. We also found that anti-social personality disorder increased the risk for PTB. However, although we also found an association between anti-social personality disorder and PTB, these results are not conclusive due to the few cases we had in our sample. Ultimately, it must be noted that these findings could not be merely explained by neglected prenatal care, lower gestational weight gain, smoking, or drinking – as previously thought. It must be noted that although we did not find any effects from depression, this may be due to the high rates of co-morbidity in this sample – among mood disorders 37.9% of women also exhibited anxiety, in comparison to just 7.1% in the group with no mood disorder.

The mechanisms through which violence and mental disorders affect birth outcomes are not clear. Violence might have a direct impact on foetal growth through trauma, by indirectly causing low weight gain and/or increasing smoking and drinking during pregnancy. It is known, however, that female victims of sexual violence are more likely to have sexually transmitted diseases and urinary tract infections, both of which cause impaired foetal growth [44]. Similarly, poor mental health may affect the infant outcome by leading to poor self-care, such as poor appetite or lesser access to antenatal services. However, our findings seem to contradict this and suggest that pathways beyond maternal behaviours may contribute more to the outcomes.

Biological pathways may explain our findings. For example, the mechanisms underlying both of these exposures and physiological alterations may involve epigenetically-mediated changes in gene expression. Extensive animal studies have demonstrated that the association between maternal psychosocial stress and low birth weight is mediated by changes in the hypothalamic-pituitary-adrenal (HPA) axis. There is also increasing evidence in humans that the HPA axis is in overdrive in pregnant women subjected to psychosocial stress [45]. In these cases, cortisol crosses the placenta, which has been demonstrated to inhibit intrauterine growth when present in high levels [46, 47]. In addition, measures of psychosocial stress, even in utero, are correlated with oxidative stress, inflammation, and telomere length. Therefore, risk factors may act through common biological pathways yielding a common phenotype: low birth weight [48]. A recent work summarized possible mechanisms that could explain the biological pathway behind growth impairment in the offspring of women exposed to psychosocial stress [49].

Higher rates of impaired foetal growth in developing countries might be explained by a higher prevalence of risk factors for violence during pregnancy, such as, poor education, adolescent pregnancy, unplanned pregnancy, low social support, relationships encouraging alcohol and drugs usage, and easy access to weapons. Moreover, in some of these countries, social and cultural norms around masculinity may endorse gendered power relationships and violence [50]. It must be noted that there may also be potential cultural biases in the various instruments used to measure violence that may skew the results.

It is important to establish if there is a common biological mechanism mediating the association between violence and impaired foetal growth, and mental disorder and impaired foetal growth. If the gestational cortisol axis were the final common pathway in the association between psychosocial stress during pregnancy and negative birth outcomes, the cortisol stress system could be a potential target for therapeutic intervention in vulnerable women. Furthermore, it is also important to establish whether the subjective experience of stress (i.e., mental disorder) in association with violence will result in relatively greater overdrive of the cortisol stress axis than either risk factor alone: i.e., whether the effects of violence and depression are additive on HPA axis measures. If there existed a cumulative effect of these risk factors, it would help in the future to identify high-risk groups.

Our study should be understood in the context of its limitations. We had a 13.1% loss of mothers in our follow up. Even if there was no statistical difference in the descriptive variables among the followed up and lost women, those who have higher rates of migration could have also had greater health risks. It must also be noted that our measurement of violence encompassed the last 7 months of gestation and a pre-conception period, making a total of 12 months. During this period, we conducted routine data collections for our outcome variables. If any measurement bias were introduced during this period, it would not be a differential bias, since exposed and non-exposed groups were submitted to similar neonatal procedures.

## Conclusion

In conclusion, domestic violence against women and mental disorders amongst pregnant women have detrimental effects on birth outcomes and unfortunately, are extremely prevalent in under-resourced, urban areas. It is imperative that actions be taken to prevent violence and improve mental health during pregnancy, especially in disadvantaged populations that may be more at risk.

## Additional files

Additional file 1: Table S1: Description of the followed up sample of pregnant women from the Butantan birth cohort. (DOCX 14 kb)

## Abbreviations

ABEP: Brazilian association of population studies
BL: Birth length
BMI: Body mass index
BW: Birth weight
DV: Domestic violence
HPA: Hypothalamic-pituitary-adrenal
ICD: International classification of diseases
IRR: Incidence rate ratio
LBW: Low birth weight
MD: Mental health disorder
OCD: Obsessive–compulsive disorder
OR: Odds ratio
PTB: Preterm birth
SGA: Small for gestational age
WHO: World Health Organization

## References

[1] Gluckman PD, Hanson MA, Bateson P, Beedle AS, Law CM, Bhutta ZA, Anokhin KV, Bougneres P, Chandak GR, Dasgupta P (2009). Towards a new developmental synthesis: adaptive developmental plasticity and human disease. Lancet.

[2] Fall CH (2013). Fetal programming and the risk of noncommunicable disease. Indian J Pediatr.

[3] Gluckman PD, Hanson MA (2004). Living with the past: evolution, development, and patterns of disease. Science.

[4] Ferrari AA, Solymos GM, Castillo RM, Sigulem DM (1998). Risk factors for protein-energy malnutrition in pre-school shantytown children in São Paulo, Brazil. Sao Paulo Med J.

[5] Patel V, Rahman A, Jacob KS, Hughes M (2004). Effect of maternal mental health on infant growth in low income countries: new evidence from South Asia. BMJ.

[6] Andersson L, Sundstrom-Poromaa I, Wulff M, Astrom M, Bixo M (2004). Neonatal outcome following maternal antenatal depression and anxiety: a population-based study. Am J Epidemiol.

[7] Chung TK, Lau TK, Yip AS, Chiu HF, Lee DT (2001). Antepartum depressive symptomatology is associated with adverse obstetric and neonatal outcomes. Psychosom Med.

[8] Rahman A, Iqbal Z, Bunn J, Lovel H, Harrington R (2004). Impact of maternal depression on infant nutritional status and illness: a cohort study. Arch Gen Psychiatry.

[9] Murphy CC, Schei B, Myhr TL, Du MJ (2001). Abuse: a risk factor for low birth weight? A systematic review and meta-analysis. CMAJ.

[10] Paarlberg KM, Vingerhoets AJ, Passchier J, Dekker GA, Van Geijn HP (1995). Psychosocial factors and pregnancy outcome: a review with emphasis on methodological issues. J Psychosom Res.

[11] Victora CG, Barreto ML, do Carmo Leal M, Monteiro CA, Schmidt MI, Paim J, Bastos FI, Almeida C, Bahia L, Travassos C, Reichenheim M, Barros FC, Lancet Brazil Series Working Group (2011). Health conditions and health-policy innovations in Brazil: the way forward. Lancet.

[12] Williams RL, Creasy RK, Cunningham GC, Hawes WE, Norris FD, Tashiro M (1982). Fetal growth and perinatal viability in California. Obstet Gynecol.

[13] Capurro H, Konichezky S, Fonseca D, Caldeyro-Barcia R (1978). A simplified method for diagnosis of gestational age in the newborn infant. J Pediatr.

[14] Garcia-Moreno C, Jansen HA, Ellsberg M, Heise L, Watts CH et al. Prevalence of intimate partner violence: findings from the WHO multi-country study on women's health and domestic violence. Lancet. 2006;368(9543):1260–9. 10.1016/S0140-6736(06)69523-8 17027732

[15] Sheehan DV, Lecrubier Y, Sheehan KH, Amorim P, Janavs J, Weiller E, Hergueta T, Baker R, Dunbar GC (1998). The Mini-International Neuropsychiatric Interview (M.I.N.I.): the development and validation of a structured diagnostic psychiatric interview for DSM-IV and ICD-10. J Clin Psychiatry.

[16] Schraiber LB, Latorre Mdo R, França I, Segri NJ, D’Oliveira AF (2010). Validity of the WHO VAW study instrument for estimating gender-based violence against women. Rev Saude Publica.

[17] Amorim, P. Mini International Neuropsychiatric Interview (MINI): validação de entrevista breve para diagnóstico de transtornos mentais. Rev Bras Psiquiatr 2000, vol.22, n.3, pp. 106-115.

[18] World Health Organization – WHO, 2001. URL: http://www.who.int/gender/violence/womenfirtseng.pdf. Accessed 14 May 2014.

[19] ABEP. Criterio de Classificaçao Econômica Brasil. Associacao Brasileira de Estudos Populacionais. Sao Paulo: ABEP; 2007.

[20] Olsen J, Frische G (1993). Social differences in reproductive health. A study on birth weight, stillbirths and congenital malformations in Denmark. Scand J Soc Med.

[21] Textor J, Hardt J, Knüppel S (2011). DAGitty: a graphical tool for analyzing causal diagrams. Epidemiology.

[22] Krug EG (2002). World report on violence and health.

[23] Cardia N. Urban violence in Sao Paulo. Washington D.C.: Woodrow Wilson International Centre for Scholars (Comparative Urban Studies Occasional Paper Series, 33), 2000.

[24] Sanjuan AN, Pinheiro PS (1998). [Youth and violence in Caracas – Paradox of a process of citizenship loss]. [Portuguese]. Sao Paulo without fear – a diagnose of urban violence.

[25] World Health Organisation (2013). Global and regional estimates of violence against women: prevalence and health effects of intimate partner violence and non-partner sexual violence.

[26] Howard LM, Oram S, Galley H, Trevillion K, Feder G (2013). Domestic violence and perinatal mental disorders: a systematic review and meta-analysis. PLoS Med.

[27] Gazmararian JA, Lazorick S, Spitz AM, Ballard TJ, Saltzman LE, Marks JS (1996). Prevalence of violence against pregnant women. [erratum appears in JAMA 1997 Apr 9;277(14):1125]. JAMA.

[28] Valdez-Santiago R, Sanin-Aguirre LH (1996). Domestic violence during pregnancy and its relationship with birth weight. Salud Publica de Mexico.

[29] Moraes CL, Reichenheim ME (2002). Domestic violence during pregnancy in Rio de Janeiro, Brazil. Int J Gyn Obst.

[30] Bennett HA, Einarson A, Taddio A, Koren G, Einarson TR (2004). Prevalence of depression during pregnancy: systematic review. [erratum appears in Obstet Gynecol. 2004 Jun;103(6):1344]. Obstet Gynecol.

[31] Heron J, O'Connor TG, Evans J, Golding J, Glover V, The ALSPAC Study Team (2004). The course of anxiety and depression through pregnancy and the postpartum in a community sample. J Affect Disord.

[32] Fatoye FO, Adeyemi AB, Oladimeji BY (2004). Emotional distress and its correlates among Nigerian women in late pregnancy. JObstetrics Gynaecol.

[33] Lovisi GM, López JR, Coutinho ES, Patel V. Poverty, violence and depression during pregnancy: a survey of mothers attending a public hospital in Brazil. Psychol Med. 2005;35(10):1485–92. 10.1017/S0033291705005362 16164772

[34] Golding JM (1999). Intimate partner violence as a risk factor for mental disorders: a meta-analysis. J Fam Violence.

[35] Ferri CP, Mitsuhiro SS, Barros MC, Chalem E, Guinsburg R, Patel (2007). The impact of maternal experience of violence and common mental disorders on neonatal outcomes: a survey of adolescent mothers in Sao Paulo, Brazil. BMC Public Health.

[36] Harpham T (1994). Urbanization and mental health in developing countries: a research role for social scientists, public health professionals and social psychiatrists. Soc Sci Med.

[37] Browm EW, Harris TO (1978). Social origins of depression.

[38] Hoffman S, Hatch MC (2000). Depressive symptomatology during pregnancy: evidence for an association with decreased fetal growth in pregnancies of lower social class women. Health Psychol.

[39] Patel V, Prince M (2006). Maternal psychological morbidity and low birth weight in India. Br J Psychiatry.

[40] Valladares ME. Low birth weight newborns and domestic violence: case-control study. Leon, Nicaragua, Universidad Nacional Autonoma de Nicaragua, 1996. Source ID: PIP/143376.

[41] Leung WC, Wong YY, Leung TW, Ho PC (2001). Pregnancy outcome following domestic violence in a Chinese community. Int J Gynaecol Obstet.

[42] Jejeebhoy SJ (1998). Associations between wife-beating and fetal and infant death: impressions from a survey in rural India. Stud Fam Plan.

[43] Menezes TC, Amorin MMR, Santos LC, Faundes A (2003). [Domestic physical violence and pregnancy: results of a survey in the postpartum period] [Portuguese]. RBGO.

[44] Campbell JC (2002). Health consequences of intimate partner violence. Lancet.

[45] O’Keane V, Scott J (2005). From ‘obstetric complications’ to a maternal-foetal origin hypothesis of mood disorder. Br J Psychiatry.

[46] Gitau R, Cameron A, Fisk N, Glover V (1998). Fetal exposure to maternal cortisol. Lancet.

[47] French NP, Hagan R, Evans SF, Godfrey M, Newnham JP (1999). Repeated antenatal corticosteroids: size at birth and subsequent development. Am J Obstet Gynecol.

[48] Newsome CA, Shiell AW, Fall CHD, Phillips DIW, Shier R, Law CM (2003). Is birth weight related to later glucose and insulin metabolism? — a systematic review. Diabet Med.

[49] Entringer S, Buss C, Wadhwa PD (2010). Prenatal stress and developmental programming of human health and disease risk: concepts and integration of empirical findings. Curr Opin Endocrinol Diabetes Obes.

[50] Naved RT, Huque H (2011). Men’s attitudes and practices regarding gender and violence against women in Bangladesh. Preliminary findings.

