# Supplementary material for: The specific and combined role of domestic violence and mental health disorders during pregnancy on new-born health
Source: BMC Pregnancy Childbirth. 2017 Aug 1;17:257. doi: 10.1186/s12884-017-1438-x (PMC5540537; doi:10.1186/s12884-017-1438-x)
Supplement: Supplementary file 1 — Description of the followed up sample of pregnant women from the Butantan birth cohort. (DOCX 14 kb) [file 12884_2017_1438_MOESM1_ESM.docx]

**Table S1**: Description of the followed up sample of pregnant women from the Butantan birth cohort

|  |  | **Studied (n=775)** | **Lost (n=117)** | **x2 test (p)** |
| --- | --- | --- | --- | --- |
| **Variable** | **Category** | **% (95%CI)** | **% (95%CI)** |  |
| Paternal occupation | Non-manual | 17.6 (14.8-20.3) | 15.3 (8.5-22.1) | 0.664 |
|  | Qualified manual | 18.4 (15.6-21.2) | 16.2 (9.3-23.2) |  |
|  | Nonqualified manual | 64.1 (60.6-67.6) | 68.5 (59.7-77.2) |  |
| Family Economic Class | A+B | 17.9 (15.2-20.6) | 13.7 (7.4-20.0) | 0.364 |
|  | C | 65.3 (61.9-68.6) | 71.8 (63.5-80.1) |  |
|  | D+E | 16.8 (14.1-19.4) | 14.5 (8.0-21.0) |  |
| Maternal Schooling | < 8 year | 18.9 (16.1-21.6) | 19.7 (12.3-27.0) | 0.204 |
|  | 8-10 years | 38.6 (35.2-42.1) | 46.2 (37.0-55.3) |  |
|  | 11 or + years | 42.5 (39.0-46.0) | 34.2 (25.5-42.9) |  |
| Adolescent Childbearing | - | 21.8 (18.9-24.7) | 25.6 (17.3-33.7) | 0.353 |
| Maternal Migration | - | 44.2 (40.1-47.7) | 40.5 (31.4-49.6) | 0.461 |
| Primipara | - | 46.9 (43.4-50.4) | 53.0 (43.8-62.2) | 0.219 |
| Unwanted pregnancy | - | 19.3 (16.5-22.1) | 25.6 (17.6-33.7) | 0.111 |
| Smoking in pregnancy | - | 16.0 (13.4-18.6) | 20.5 (13.1-27.9) | 0.226 |
| Drinking in pregnancy | - | 9.4 (7.4-11.5) | 14.5 (8.0-21.0) | 0.088 |
